# Spatial Optimization to Improve COVID-19 Vaccine Allocation

**DOI:** 10.3390/vaccines11010064

**Published:** 2022-12-28

**Authors:** Stephen Scroggins, Justin Goodson, Tasnova Afroze, Enbal Shacham

**Affiliations:** 1 Taylor Geospatial Institute, College for Public Health and Social Justice, Saint Louis University, St. Louis, MO 63103, USA; 2 Department of Operations and IT Management, Chaifetz School of Business, Saint Louis University, St. Louis, MO 63103, USA

**Keywords:** delivery of health care, vaccines, ecological and environmental COVID-19

## Abstract

Early distribution of COVID-19 vaccines was largely driven by population size and did not account for COVID-19 prevalence nor location characteristics. In this study, we applied an optimization framework to identify distribution strategies that would have lowered COVID-19 related morbidity and mortality. During the first half of 2021 in the state of Missouri, optimized vaccine allocation would have decreased case incidence by 8% with 5926 fewer COVID-19 cases, 106 fewer deaths, and 4.5 million dollars in healthcare cost saved. As COVID-19 variants continue to be identified, and the likelihood of future pandemics remains high, application of resource optimization should be a priority for policy makers.

## 1. Introduction

The COVID-19 pandemic has had a devastating effect worldwide. In the U.S. alone, by the end of 2020 there were more than 20 million reported infections, greater than 1.1 million related hospitalizations, and nearly 364,000 related deaths [1]. As demand for beds, medical personnel, and equipment quickly outpaced supply, hospitals turned away the ill and suspended preventive and elective procedures [2,3]. While multiple COVID-19 vaccinations were developed at unprecedented speeds and made available to general adult U.S. populations by early 2021, challenges in the distribution of limited vaccine supplies quickly arose [4,5,6].

In the U.S., states received the bulk of vaccine supplies from the federal government in an amount typically proportional to their population size [7]. Each state was tasked with downstream distribution to residents and local agencies. Although consumer vaccine distribution in most states was preliminarily undertaken in phases, based on infection susceptibility and likelihood [8,9], this, and subsequent distribution to the general adult population, was largely based on geographic population size. Though a local distribution method based on population size appealed to a sense of equality, it negated a typically more accepted needs-based approach. Currently, the possibility of optimal vaccine allocation during the early stages of a pandemic is not well understood.

Due to the infectious mode of respiratory person-to-person transmission, location and population mobility continue to play a key role in the spread of COVID-19 [10]. Population mobility patterns inform risk of infectious disease exposure as well as highlight the varying and local non-pharmaceutical prevention mandates implemented during the pandemic. These mandates included limited business hours, reduced public location capacity, and stay-at-home orders. The time populations spend at locations where people are likely to interact—such as restaurants, health provider offices, grocery stores, and religious institutions or places of worship—are a dominant factor that has shaped this pandemic [11,12,13,14]. These factors need to be included in disease spread prediction models, and further, guide vaccination distribution methods. Integrating classical models of infectious disease transmission into a vaccine allocation optimization framework is challenging. In typical compartmental models, the equations governing transitions among classes are non-convex. Incorporating these transitions into an optimization model results in computationally challenging problems. In most cases, the allocations returned by state-of-the-art optimization techniques carry no guarantee of optimality [9]. Further, the complexity of such methods restricts their accessibility and use in practice, an important requirement as COVID-19 continues to spread, causing morbidity and mortality.

The purpose of this study was to identify optimal spatial allocation methods for COVID-19 vaccines with the objective of minimizing reported COVID-19 cases. Utilizing GPS data from smart devices, we incorporated mobility and location factors into a mixed-effect Poisson model predicting the spread of COVID-19 infections. When placed in an optimization framework, the resulting model is a convex math program, readily solvable through widely available software. By showing how to overcome major obstacles to better vaccine allocation, the methods we propose are both timely and practical.

## 2. Materials and Methods

### 2.1. Sample

This study utilized an econometric, repeated measure design with the 115 counties comprising Missouri as subjects, each with 26 weekly observations from January 2021 to July 2021. This study period was chosen to coincide with recommendation and release of vaccines to adult residents [8]. While this study was geographically limited to the state of Missouri, the location provides good insight to the patterns that were occurring in other states as well: having an urban and rural composition and diverse non-pharmaceutical COVID-19 mitigation strategies during this period of time across some counties in the state. Data used for the study were collected from three primary sources. 

### 2.2. Measures

To build and assess vaccine allocation scenarios, the most appropriate outcome variable for this study was new weekly reported cases of COVID-19 per county, collected from the Missouri Department of Health and Senior Services [15]. Weekly observations, rather than daily case counts, limited day-of-the-week reporting bias and more readily included retroactive data corrections. 

Weekly vaccine uptake, for all available manufactured vaccines, among county residents was used as the study’s primary predictor and was collected from publicly available Missouri data [16]. COVID-19 vaccine uptake was divided by two to reflect the two-dose vaccine requirement needed to reach recommended immunological protection [15].

Aggregated and anonymized GPS data were collected from the data management firm Safegraph, LLC. This mobility data consisted of a rotating sample of 5–6% of the U.S. population who have consented to share data detailing time and location of visits outside the home [17]. The data were stratified according to county of residence and then temporally across types of locations visited. Locations were organized by the North American Industrial Classification System. Approximately 250 GB of uncompressed data were extracted from Safegraph, LLC prior to cleaning, organizing, and aggregating on the county level. We leveraged prior research to identify locations where risk of COVID-19 exposure would likely increase. These locations included restaurants/bars, health provider offices, grocery stores, education facilities, senior living facilities, retail locations, and religious institutions [11,12,13,14]. Additional details regarding mobility data collection have been published elsewhere [18,19].

We also estimated the number of COVID-19-related deaths and hospital costs related to COVID-19 for use in analysis. These figures were calculated by using the number of new COVID-19 cases along with the average national COVID-19 case fatality rate, average national rate of hospitalizations due to COVID-19 infections, and hospital treatment costs of COVID-19 at time of respective observations [1,20].

#### Statistical Analysis and Optimization

Statistical analysis of the data was completed in three phases. First, descriptive statistics identified temporal trends and variability of COVID-19 infections across Missouri counties. Second, a mixed-effect generalized linear regression characterized the temporal correlation between COVID-19 vaccine distribution and COVID-19 case counts. The number of new COVID-19 cases was fit with a Poisson distribution to accommodate the non-negative count nature of the model outcome. A random effect was added to account for the nested nature of observations within the 115 counties. Fixed effects included average time spent at grocery stores, restaurants and bars, retail stores, healthcare delivery and service locations, education facilities, and senior living facilities per week per resident to reflect the variation in risk inherent among these locations. Further, we included the average distance traveled when residents visited locations outside of their home. Lastly, we included the estimated total population of each county.

In the last phase of this analysis, the regression model was combined with a prescriptive optimization model for vaccine allocation. Given a limited supply of vaccines arriving across the study period, we allocated doses to Missouri counties such that the expected number of infections was minimized. The optimization utilized the Poisson model of disease spread to forecast case prevalence. We detail the optimization model and optimization software used in the Supplemental Material [21].

To depict the effects of optimization, we devised nine scenarios by varying proportions of actual vaccine supply and resident mobility (time and distance traveled). Each scenario held supply and mobility factors at their actual levels, reduced them by 50%, or increased them by 100%. These scenarios provided different sets of mobility data and supply schedules to the optimization, but held the fixed and random effects constant. In addition to identifying the minimum number of expected new COVID-19 cases in each scenario, we connected these figures to the expected number of COVID-19 related deaths and to expected hospital costs.

## 3. Results

**Figure 1:**
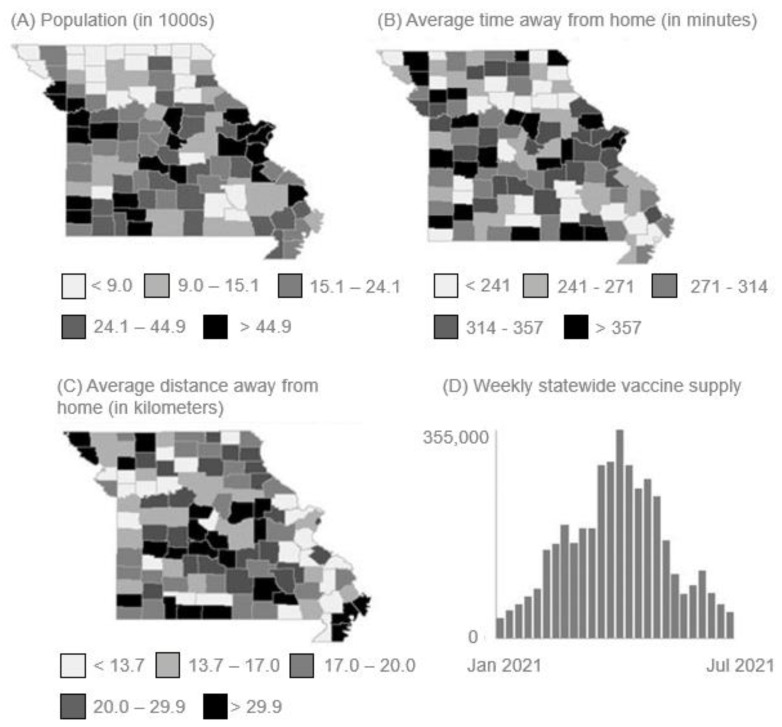
Descriptive quantile maps of (**A**) Missouri county populations (as of 2019), (**B**) average visit time (minutes) spent at a location outside the home (Jan 2021–July 2021), (**C**) average distance (kilometers) residents traveled to visit a location outside the home (Jan 2021–July 20201), (**D**) weekly number of COVID-19 vaccines distributed among Missouri counties (Jan 2021–July 2021).

Across the 115 counties of Missouri, there are 6,154,913 residents. Of those, 22.4% (*n* = 1,378,701) are under 18 years of age, 54.2% (*n* = 3,335,963) are 19-64 years of age, and 17.6% (*n* = 1,083,264) are older than 65 years. The population size for each county is depicted in Figure 1A. The majority of the state’s residents are white, while 11.8% (*n* = 726,280) identify as Black/African American and 4.7% (*n* = 289,281) as Hispanic/Latino.

During the study period, a total of 173,656 COVID-19 cases were reported among Missouri counties for an average of 58.7 cases per week per county (SD 220.2). At the end of the study (July 2021), counties across the state had an average vaccination rate of 26.8% (SD 7.3%). Residents spent an average of 113.4 min (SD 64.2) when visiting senior living facilities, 99.4 min (SD 54.3) at healthcare facilities, 87.3 min (SD 26.2) at educational facilities, 43.1 min (SD 63.1) at grocery and food stores, 38.5 min (SD 16.1) at retail locations, and 37.2 min (SD 17.4) at restaurants and bars. Overall, residents traveled an average of 21.8 km (SD 13.3) to reach these locations during the study period. 

Differences in county population sizes are depicted in the quantile map in Figure 1A. The quantile maps in Figure 1B and Figure 1C show differences in average time at the specified locations and average distance traveled, respectively. Figure 1D displays the number of vaccines distributed among all counties at each week of the study, peaking at 354,894 during week 14. 

Results of the mixed-effect regression, which was designed to predict number of COVID-19 cases, are detailed in Table 1 with the estimated variable coefficients expressed in a log link response. Each variable included in the model was shown to be significantly associated with the response variable. The cumulative percent of vaccinated individuals increased and the number of new COVID-19 cases decreased across the state significantly. COVID-19 case rates increased significantly as time spent at any commercial locations was documented. Each county’s population and their higher average distance traveled away from home was significantly associated with to higher case counts.

**Figure 2:**
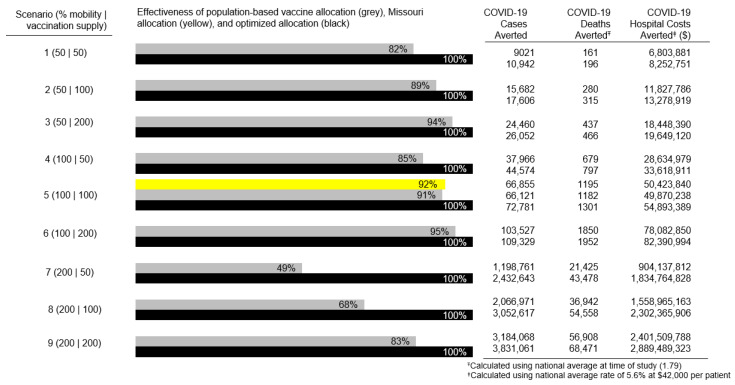
Effectiveness of population-based vaccine allocation and Missouri state allocation compared to spatially optimized allocation under 9 different scenarios of varying geographic mobility.

For each of the nine mobility-supply scenarios, Figure 2 compares the performance of optimal allocation against a population-based allocation, where vaccines were distributed only according to population size. In the 100% mobility and 100% supply scenario (Figure 2, Scenario 5), the state of Missouri’s actual allocation policy was used as a second benchmark. In this scenario, we predict spatial optimization of vaccine allocation would have averted 72,781 COVID-19 cases, averted 1301 COVID-19 related deaths, and saved $54,893,389 in COVID-19 related hospital costs. The optimal vaccine allocation was 9 percentage points more effective, based on averted cases, than the population-based allocation and 8 percentage points more effective than Missouri’s actual allocation. The largest disparity between optimized allocation and population-based allocation was seen when resident mobility was doubled and vaccine supply was halved (Figure 2, Scenario 7). Under these parameters, optimized allocation averted twice as many cases as the population-based allocation method. Even under the most favorable parameters, with mobility halved and vaccine supply doubled (Figure 2, Scenario 3), the number of cases averted by the optimal allocation was 6 percentage points higher than the number averted by the population-based allocation. 

**Figure 3:**
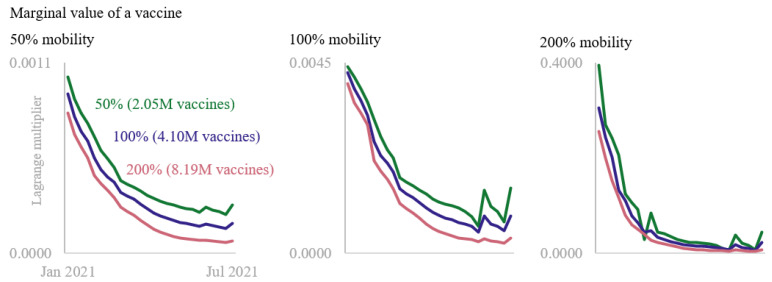
Temporal value of COVID-19 vaccines from January 2021–July 2021 under differing mobility and supply scenarios.

Finally, we examined the value of vaccines across time in an optimal allocation policy. For each of the 9 scenarios, Figure 3 displays the dual variables associated with weekly supply constraints (3). Due to large differences in dual values, we display the figures in three charts with identical horizontal scales but with different vertical scales for each mobility level (50%, 100%, 200%). A value in Figure 3 can roughly be interpreted as the decrease in case count that would have resulted from one additional vaccine available for allocation during a particular week. This information is a unique byproduct of mathematical optimization and cannot be obtained through other means.

## 4. Discussion

The purpose of this study was to understand the impact of spatially optimal COVID-19 vaccine allocation in Missouri. Results suggest optimal allocation would have markedly improved health outcomes, reducing the number of cases by 8% during a 6 month period of time. These findings suggest that including variables that increase risk of an infectious disease more accurately will reduce morbidity and mortality. While mobility data had not been widely used prior to the COVID-19 pandemic to inform public health and healthcare efforts, they have been found to be especially useful in predicting a respiratory infectious disease. 

This study also found that across all scenarios in Figure 3, vaccines were generally more valuable when they were allocated earlier rather than later. For example, when mobility was 50% and supply was 200% (Figure 2, Scenario 3), an additional vaccine had more than 12 times the impact in early January 2021 than it would have had toward the end of June 2021. This difference increased to more than 73 times when mobility was 200% and supply was 50% (Figure 2, Scenario 7). Because COVID-19 infections grew at an exponential rate, a given number of vaccines was more effective at slowing disease spread in the early parts of a pandemic than the same amount would have been later. The kinks in each series are related to variations in the supply schedule. When the number of vaccines available for allocation during a particular week was lower than the supply the week before and the week after, additional vaccines during that week were more valuable.

Second, across all time periods, the value of a vaccine increased substantially as mobility of the population increased. For instance, when supply was at 50%, the dual variable corresponding to the week-one supply constraint increased by more than 4 times when mobility moved from 50% to 100%, then by an additional 90 times when mobility increased to 200 percent. That is more than a 38,000% increase from low to high mobility. This enormous difference points to the importance of mobility in curbing a pandemic. It is not that a vaccine’s ability to inoculate somehow increases as individuals spend more time outside of their residences and venture further away from their homes. Rather, as Figure 2 shows, the number of infections to be averted is orders of magnitude higher, and thus the potential for a vaccine to decrease disease spread is also much higher.

While other optimization models primarily utilized population age as a means to allocate vaccines [22,23,24], this study relies on mobility in rural, suburban, and urban communities. Including these factors in the vaccine optimization model allows for stronger predictive inputs that inform the output far more than the allocation method used in Missouri, and in many other states at the time of allocation. Though the literature on COVID-19 vaccine allocation is young, the same realism-tractability challenge faced by many fields is present here. Optimization models that integrate location, mobility, and disease dynamics are better representations of reality than those that do not, but they are significantly more difficult to solve [24,25]. 

Our work considered the roles of mobility and location in disease progression and also provided guidance for optimal vaccine allocation policies. We demonstrated how optimal policies could have averted infections, deaths, and hospital costs in the Missouri during the first half of 2021, a period when vaccine supplies were low, and COVID-19 infections continued to increase. Across a range of scenarios, we showed the potential for an optimal allocation of vaccines to improve upon policies based on population size. We found that the benefits of optimal allocation increased dramatically in scenarios with higher mobility and fewer vaccines. However, even when mobility was low, and supplies were more abundant, optimal allocation of vaccines still led to reductions in case rates, fatalities, and hospital costs. 

To conceptualize findings and propel future research, several study limitations were identified. Due to data availability, this study worked under the assumption that distribution of vaccines equated to administration of vaccines. However, news sources revealed that at times vaccines go unused [26,27]. In addition to including geographic mobility, it may be beneficial for future studies to consider collective community beliefs and attitudes surrounding likelihood of vaccine uptake. While this study also gives an estimate of COVID-19 deaths and hospitalization costs, these values are based on national averages and, like infection rates, are likely a product of geographic variation [28,29]. Further, infections may have been unreported during the study period. Additional studies would benefit from deeper examination of these variables and the role they play in optimal vaccine allocation policies. 

Our work provides an important public health tool for the future. In the face of new COVID-19 variants, our analysis can be used to guide the distribution of limited supplies of resources, as well as to prioritize communities that may be affected earlier than others due to mobility. Further, as we prepare for the possibility of other pandemics, this research lays a foundation for the integration of important environmental factors into predictive disease models and prescriptive optimization tools. 

## Figures and Tables

**Table 1:** Fixed effect estimates predicting weekly COVID-19 cases across Missouri Counties from January 2021 to July 2021.

	Coefficient Estimate ^†^	95% Confidence Interval	*p*-Value
% Vaccinated	−2.488	−2.552, −2.423	<0.001
Time spent at grocery/food stores (min)	0.001	0.000, 0.001	<0.001
Time spent at restaurants/bars (min)	0.014	0.013, 0.015	<0.001
Time spent at retail locations (min)	0.016	0.015, 0.016	<0.001
Time spent at healthcare locations (min)	0.004	0.003, 0.004	<0.001
Time spent at education locations (min)	0.001	0.011, 0.012	<0.001
Time spent at senior living facility (min)	0.004	0.004, 0.004	<0.001
Distance traveled from residence (km)	0.371	0.356, 0.385	<0.001
Population ^‡^	0.912	0.709, 1.114	<0.001

^†^ log link response, ^‡^ scaled around minimum and maximum values.

## Data Availability

Disease prevalence data utilized in this study was collected from publicly available disease surveillance data published by The Missouri Department of Health and Senior Services. Geographic placed-based data is available from Safegraph LLC as part of an academic research consortium.

## Supplementary Materials

The following supporting information can be downloaded at: https://www.mdpi.com/article/10.3390/vaccines11010064/s1, S1: Proof of Convexity.
