# Supplementary material for: Spatial Optimization to Improve COVID-19 Vaccine Allocation
Source: Vaccines (Basel). 2022 Dec 28;11(1):64. doi: 10.3390/vaccines11010064 (PMC9866695; doi:10.3390/vaccines11010064)
Supplement: Supplementary file 1 [file vaccines-11-00064-s001.zip › vaccines-2059726-supplementary.pdf]

vaccines

Article

Spatial Optimization to Improve COVID-19 Vaccine Allocation

Stephen Scroggins [1], Justin Goodson [2], Tasnova Afroze [2] and Enbal Shacham[1,*]

[1] Taylor Geospatial Institute and College for Public Health and Social Justice, Saint Louis University, St. Louis, MO 63103, USA
[2] Department of Operations and IT Management, Chaifetz School of Business, Saint Louis University, St. Louis, MO 63103, USA
* Correspondence: enbal.shacham@slu.edu

Supplemental Material

Here we detail how the regression model was combined with a prescriptive optimization model for vaccine allocation. Given a limited supply of vaccines arriving across weeks 1, 2, . . ., T=26, we allocated doses such that the expected number of infections across counties 1, 2, . . ., K=115 was minimized. During each week t, we chose v_t^k, a number between zero and one representing a proportion of the population in county k. Letting N_k be the population size at county k, we allocated $N^k v_t^k$ vaccines to county k during week t. We let d_t be the number of doses scheduled to arrive at the beginning of week t and assumed leftover inventory of vaccines could be carried from one week to the next.

The optimization utilized the Poisson model of disease spread to forecast case prevalence. We linked decision variables to the regression equation via the function $v^k(t) = \sum_{t'=1}^{t} v_{t'}^k$, the proportion of the population at location k vaccinated from the beginning of the study period through week t. The optimization model utilized the above-described covariates and their effects. Let $\log E\,[Y_{tk}|x_{tk}] = a_{tk} + \beta v^k(t)$ be the Poisson regression equation predicting the log of the expected number of cases Y_{tk} during week t in county k given covariate vector x_{tk}, where β is the vaccination effect and a_{tk} represents the fixed and random effects associated with the remaining covariates. The predicted number of cases was obtained by exponentiation.

The vaccine allocation problem was modeled as the following math program:

Minimize:
$$f(x) = \sum_{t=1}^{T}\sum_{k=1}^{K} \exp\left(a_t^k + \beta v^k(t)\right) \quad (1)$$

Subject To:
$$2\sum_{t'=1}^{t}\sum_{k=1}^{K} N_k v_{t'}^k \le \sum_{t'=1}^{t} d_{t'}\,,\ t = 1,\ 2, \dots, T \quad (2)$$

$$v^k(T) \le 1, k = 1,2, \dots, K \quad (3)$$

$$v_t^k \ge 0, t = 1,2, \dots, T, \qquad k = 1,2, \dots, K \quad (4)$$

The objective function (1) was the expected total number of infections across all weeks and locations. Constraints (2) limited vaccine allocation in week t to the total number of vaccines available in that week, which consisted of new deliveries plus any vaccines not allocated during previous weeks. Constraints (2) also required two vaccinations per individual to be fully immunized. Constraints (3) limited the vaccines allocated to each county to be less than or equal to the county's population. Constraints (4) required non-

negative allocations. In practice, when $N_k v_t^k$ vaccines is not a whole number, then rounding down to the nearest integer resulted in a feasible allocation.

We solved all instances of problem (1)-(4) with the Knitro solver. Because the math program is convex, any locally optimal allocation will also be a globally optimal allocation, meaning a better allocation does not exist [9]. To show that problem (1)-(4) is a convex program, the objective function must be a convex function and the constraints must form a convex set. As the constraints are all linear, they trivially form a convex set. We demonstrate that objective function *f* is convex by showing that the Hessian matrix, which contains all second partial derivatives of *f,* is positive definite. We begin with first partial derivatives. The partial derivative of *f* with respect to v_t^k is

$$\frac{\partial f}{\partial v_t^k} = \sum_{\tilde{t}=t}^{T} \beta\, exp\left(a_{\tilde{t}}^k + \beta v^k(\tilde{t})\right).$$

Further differentiating with respect to $v_{\bar{t}}^{\bar{k}}$ gives

$$\frac{\partial^2 f}{\partial v_t^k \partial v_{\bar{t}}^{\bar{k}}} = \begin{cases} \sum_{\tilde{t}=\max\{\bar{t},t\}}^{T} \beta^2\, exp\left(a_{\tilde{t}}^k + \beta v^k(\tilde{t})\right),\ k = \bar{k}, \\ 0, \text{otherwise.} \end{cases}$$

Note that the second partial derivative is strictly positive if $k = \bar{k}$, otherwise it is zero. When $k = \bar{k}$, the summation goes from the larger of t and $\bar{t}$ up to *T*. Consequently, the Hessian matrix can be constructed as a block diagonal matrix, where each block has special structure. For a given *k*, define the block

$$P_k = \begin{bmatrix} \frac{\partial^2 f}{\partial v_1^k \partial v_1^k} & \frac{\partial^2 f}{\partial v_1^k \partial v_2^k} & \cdots & \frac{\partial^2 f}{\partial v_1^k \partial v_T^k} \\ \frac{\partial^2 f}{\partial v_2^k \partial v_1^k} & \frac{\partial^2 f}{\partial v_2^k \partial v_2^k} & \cdots & \frac{\partial^2 f}{\partial v_2^k \partial v_T^k} \\ \vdots & \vdots & \ddots & \vdots \\ \frac{\partial^2 f}{\partial v_T^k \partial v_1^k} & \frac{\partial^2 f}{\partial v_T^k \partial v_2^k} & \cdots & \frac{\partial^2 f}{\partial v_T^k \partial v_T^k} \end{bmatrix}.$$

In the tth row, the first *t* elements are identical. Then, denoting by **0** the *T*-by-*T* matrix of zeros, the Hessian is

$$H(x) = \begin{bmatrix} P_1 & \mathbf{0} & \cdots & \mathbf{0} \\ \mathbf{0} & P_2 & \cdots & \mathbf{0} \\ \vdots & \vdots & \ddots & \vdots \\ \mathbf{0} & \mathbf{0} & \cdots & P_K \end{bmatrix}.$$

Objective function *f* is convex if *H* is positive definite. One way to demonstrate that *H* is positive definite is to show that its pivots are all positive. To do this, use Gaussian elimination to put *H* in echelon form by performing the following row operations on each block: for each row *t* except the last row, replace row *t* with row *t* less row *t+1*. In the new row, if $\bar{t}$>t, all terms cancel, and the result is zero. Otherwise, the result is strictly positive. It follows that each block is a lower triangular matrix of strictly positive values, and thus the pivots, which constitute the diagonal, are also positive. Thus, the block is positive definite. Performing identical operations on all blocks demonstrates that *H* is positive definite, and thus *f* is convex.
